# Long-Term Refrigerated Storage of Beef Using an Active Edible Film Reinforced with Mesoporous Silica Nanoparticles Containing Oregano Essential Oil (*Lippia graveolens* Kunth)

**DOI:** 10.3390/ijms24010092

**Published:** 2022-12-21

**Authors:** Alexis Matadamas-Ortiz, Elvia Hernández-Hernández, Eduardo Castaño-Tostado, Aldo Amaro-Reyes, Blanca E. García-Almendárez, Gonzalo Velazquez, Carlos Regalado-González

**Affiliations:** 1 Department of Food Research and Postgraduate Studies, Faculty of Chemistry, Autonomous University of Querétaro, C.U., Cerro de las Campanas s/n, Col. Las Campanas, Querétaro 76010, Mexico; 2 National Polytechnic Institute, Center for Research in Applied Science and Advanced Technology, Querétaro Unit, Cerro Blanco No. 141, Col. Colinas del Cimatario, Querétaro 76090, Mexico

**Keywords:** fresh beef preservation, active edible films, oregano essential oil, mesoporous silica nanoparticles

## Abstract

Beef is a fundamental part of the human diet, but it is highly susceptible to microbiological and physicochemical deterioration which decrease its shelf life. This work aimed to formulate an active edible film (AEF) incorporated with amino-functionalized mesoporous silica nanoparticles (A-MSN) loaded with Mexican oregano (*Lippia graveolens* Kunth) essential oil (OEO) and to evaluate its effect as a coating on fresh beef quality during refrigerated storage. The AEF was based on amaranth protein isolate (API) and chitosan (CH) (4:1, *w*/*w*), to which OEO emulsified or encapsulated in A-MSN was added. The tensile strength (36.91 ± 1.37 MPa), Young’s modulus (1354.80 ± 64.6 MPa), and elongation (4.71%) parameters of AEF made it comparable with synthetic films. The antimicrobial activity of AEF against *E. coli* O157:H7 was improved by adding 9% (*w*/*w*) encapsulated OEO, and interactions of glycerol and A-MSN with the polymeric matrix were observed by FT-IR spectroscopy. In fresh beef, after 42 days, AEF reduced the population growth (Log CFU/cm^2^, relative to uncoated fresh beef) of *Brochothrix thermosphacta* (5.5), *Escherichia coli* (3.5), *Pseudomonas* spp. (2.8), and aerobic mesophilic bacteria (6.8). After 21 days, odor acceptability of coated fresh beef was improved, thus, enlarging the shelf life of the beef and demonstrating the preservation capacity of this film.

## 1. Introduction

Fresh meat is a good source of nutrients, but it is also a complex matrix where food-related pathogens and spoilage microorganisms can easily grow. To extend the shelf life and ensure the safety of these products, storage under vacuum refrigeration or in modified atmosphere packaging (MAP) can be used. However, these conditions allow both the survival and growth of pathogenic and spoilage strains, as well as the development of oxidation reactions [1]. For consumers, red color on meat surface is a very desirable characteristic which is directly related to quality. Under vacuum conditions, beef maintains a reddish-purple color due to the formation of deoxymyoglobin; in oxygen-permeable packaging beef shows a bright red color due to formation of oxymyoglobin, but continuous exposure to oxygen favors oxidation of lipids and accumulation of metmyoglobin on meat surface, producing an undesirable brown color [2]. In addition, the oxidation of lipids produces meat rancidity which affects the characteristic flavor of the packaged meat [3]. Consequently, if oxidation reactions and microbial growth are delayed, the shelf life of meat can be enlarged. A possible alternative to solve the problem of quality loss in fresh beef cuts is the use of edible coatings that can be applied as primary package of the meat.

Edible films and coatings are thin layers of edible materials, and are based on proteins, carbohydrates, lipids, or their mixture. When applied to food products, they play an important role in their preservation, distribution, and marketing, showing benefits over synthetic films [4]. Amaranth is a pseudocereal that contains a high protein content, has a balanced amino acid composition, and is recognized as a potential source of food products [5]. It has been used to produce edible films and coatings on a laboratory scale [6]. On the other side, chitosan (CH) is the second most abundant polysaccharide in nature after cellulose, and is non-toxic and biodegradable. Its composition is based on glucosamine and N-acetylglucosamine units linked by β-(1→4) glycosidic bonds [7] and is extensively used in films and coatings production due to its ability to inhibit the growth of some pathogenic bacteria and fungi [8]. Edible films and coatings can also perform as a vehicle of molecules with antimicrobial or antioxidant activity by their incorporation within the polymeric matrix.

Essential oils (EO) are extracted from plant materials. They are considered secondary metabolites with proven antimicrobial, antiparasitic, insecticidal, antiviral, antifungal, and antioxidant properties. Their composition is complex and mainly based on aromatic and volatile compounds [9]. The spice known as Mexican oregano belongs to the *Verbenaceae* family [10]; due to their variability in chemical and aroma characteristics, *Origanum* plants are widely used as culinary herbs, flavoring substances of food products, alcoholic beverages, perfumery, and to treat various ailments in folk medicine [11]. Previous studies have shown that Mexican oregano (*Lippia graveolens* Kunth) essential oil (OEO) is effective against bacteria, yeasts, and fungi primarily due to its thymol and carvacrol content. These compounds can increase the permeability of cell membranes generating their disintegration and promoting dissipation of the proton-motive force; in addition, thymol shows an influence on genes that synthesize membrane proteins [1,12]. However, use of EO is limited because of its volatile nature and its susceptibility to temperature, light, or oxygen. Encapsulation of EO offers an alternative to increase its functional characteristics, such as the use of mesoporous silica nanoparticles (MSN) that provide protection, prevention of volatilization, stability increase, long-term effects, and miscibility in aqueous suspension [13]. MSN are silicon dioxide (SiO_2_) nanostructures that exhibit pores with sizes of 2–50 nm [14] where the EO can be lodged. Additionally, MSN can also act as reinforcer of the edible film, producing a nanobiocomposite with improved mechanical properties [15]. Several reports indicate that MSN have acceptable biocompatibility and low toxicity, especially when the particle size is above 100 nm [16].

The objective of this work was to produce an active edible film incorporating mesoporous silica nanoparticles containing oregano (*Lippia graveolens* Kunth) essential oil to preserve the physicochemical, microbiological, and sensory properties of fresh beef stored under refrigeration.

The encapsulation of oregano essential oil in MSN successfully achieved the preservation of its antioxidant and antimicrobial properties and reduced its destabilizing effect on the edible active film matrix. The designed active edible film increased the shelf life of fresh beef stored under refrigeration, inhibiting the development of pathogenic and spoilage microorganisms for 42 d, while achieving consumer acceptability for up to 21 d.

## 2. Results and Discussion

### 2.1. Amaranth Protein Isolate Production

The protein content of amaranth flour was 13.98 ± 0.17% (*w*/*w*), in agreement with other reports (Table 1). After two washes, the API protein concentration increased from 57.95% to 61.34% (*w*/*w*, dry basis), which was lower than those obtained by Das et al. [17] and Cortez-Trejo et al. [18]. These authors included a defatting step with organic solvents before milling, followed by drying of the flour, while the API was obtained by freeze drying, with consequent increase in protein concentration, given the fat (7.0–7.33) and moisture (9.51–12.4) content of the grain. In contrast, the yield of our API was notably higher, indicating good solubilization of the amaranth protein in aqueous solution at pH 9.

### 2.2. Mesoporous Silica Nanoparticles Morphology

**Figure 1:**
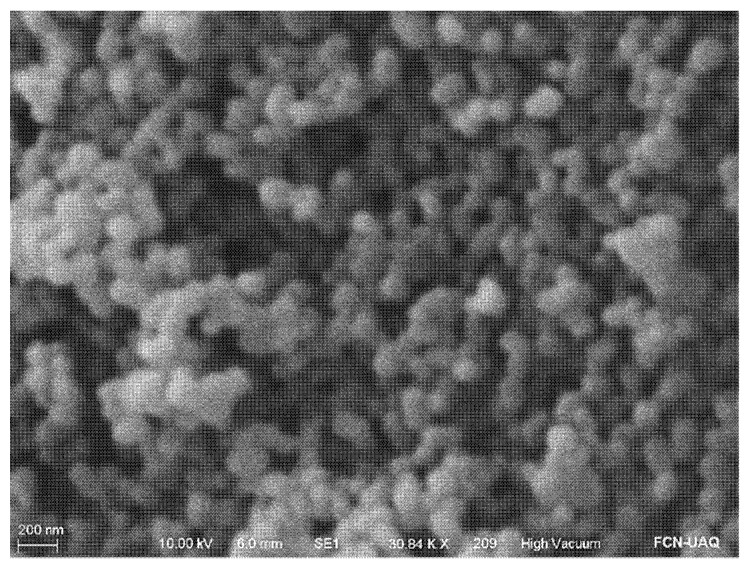
Scanning electron micrograph of amino-functionalized MSN (A-MSN). Amplification ≈ 30 kX.

Figure 1 shows circular particles of A-MSN, with an average size of 146.49 ± 20.49 nm, suggesting their safe use on food matrices, since according to Gou et al. [19], the biosafety of A-MSN is influenced by particle and pore size, shape, and possible surface modifications.

Lu et al. [20], working with HeLa cells, reported the uptake rate of MSN with particles size ranging between 30–280 nm, found that 50 nm particles showed the highest internalization rate, while the lowest was exhibited by 170 nm particles. According to these authors, smaller MSN are more suitable for carrying therapeutic agents for biological applications, but as carriers of bioactive compounds for biopreservation of food products, larger MSN size could prevent their interaction with cells. Additionally, hemolysis and cytotoxicity tests of MSN on erythrocytes have been studied [21], and it was concluded that smaller particles caused greater cell damage and higher hemolytic activity than larger particles. Furthermore, functionalized MSN modified with amine or thiol functional groups have shown lower cytotoxicity than the unmodified MSN [22].

### 2.3. Antimicrobial Effect of Oregano Essential Oil and Chitosan

#### 2.3.1. Minimum Inhibitory Concentration of Oregano Essential Oil and Chitosan against *E. coli* O157:H7

**Figure 2:**
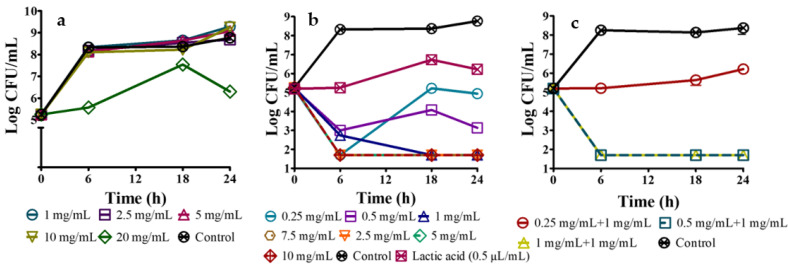
Minimum inhibitory concentration (MIC) of the different antimicrobial agents used, against *E. coli* O157:H7. (**a**) oregano essential oil (OEO); (**b**) chitosan (CH); (**c**) chitosan and oregano essential oil mixture (CH + OEO).

Complete inhibition of *E. coli* O157:H7 could not be observed for OEO concentrations used. However, the largest concentration of OEO used (20 mg/mL) resulted in a bacteriostatic effect as the population was controlled at 5.56 ± 0.07 Log CFU/mL, but this effect was lost after 18 h when the population increased 2 Log cycles (Figure 2a). The sublethal effect of OEO has been attributed to an increased concentration of saturated fatty acids of the bacterial membrane, causing a non-specific decrease in cell permeability [23,24]. On the other hand, after 6 h of incubation, the microbial population decreased in the presence of 0.25 and 0.5 mg/mL CH, but this effect only lasted for 18 h (Figure 2b). *E. coli* O157:H7 was completely inhibited after 18 h of incubation using 1 mg/mL of CH, while complete inhibition after 6 h required higher concentrations (2.5–10 mg/mL) (Figure 2b). Therefore, for *E. coli* O157:H7, the minimum inhibitory concentration (MIC) was >20 mg OEO/mL and 2.5 mg CH/mL; however, a MIC < 5 mg OEO/mL for this microorganism has been reported [1]. It should be clarified that these authors reported immediate processing of oregano leaves once harvested and dried, while in this research they were already dehydrated when obtained and stored for about six months. Hernandez-Hernandez et al. [12] reported that the inhibition of *Micrococcus luteus* by OEO was reduced by 40.7% after 3 months of refrigerated storage in amber colored vials, indicating poor stability of its components. Given these conditions, storage time is considered a significant factor in the quality of oregano leaves and its essential oil, which could also be related to the lower extraction yield in this case (3.36 ± 0.8%, *w*/*w*, d.b.) than that reported by these authors (4.29% *w*/*w*, d.b.). In addition, the composition of essential oils can be affected by a variety of factors, such as the part of the plant used for extraction, the type of drying, the maturation stage of the plant during harvest, and growing conditions such as soil type, temperature and fertilizers used [25].

On the other hand, the antimicrobial effect of CH is associated with its cationic behavior, exerting electrostatic interactions through its positively charged amino groups with the negative charge of the bacterial cell membrane, leading to cell lysis. Furthermore, CH antimicrobial activity is benefited by the characteristics of the peptidoglycan layer of Gram-negative bacteria, which shows lower thickness than Gram-positive bacteria, making them more susceptible [26]. Additionally, it should be emphasized that 0.05% *v/v* lactic acid (whose antimicrobial activity has been widely reported, Figure 2b) was used to dissolve CH resulting in a pH of 3.1, promoting a strong ionic interaction due to the protonated amino groups, causing cell wall disruption [26,27].

#### 2.3.2. Combined Minimum Inhibitory Concentration of Oregano Essential Oil and Chitosan against *E. coli* O157:H7

The combination of OEO and CH showed greater antimicrobial activity than individually. From Figure 2c, the application of 0.25 mg/mL CH plus 1 mg/mL OEO exhibited a bacteriostatic effect that was maintained for 24 h while the combination of 0.5 mg/mL CH and 1 mg/mL OEO showed a bactericidal effect for up to 24 h. It was determined that FIC_CH_ = 0.2 and FIC_OEO_ < 0.05, thus 0.20 < FIC_index_ < 0.25, indicated a synergistic effect between CH and OEO. Theoretical mechanisms of synergistic interactions of antimicrobials have been described [9], such as sequential inhibition of various steps of a biochemical pathway, inhibition of enzymes that degrade or excrete antimicrobials, and interaction with the cell wall or cell membrane, although details of these interactions are still unclear.

### 2.4. Loading Capacity of Mesoporous Silica Nanoparticles Loaded with Oregano Essential Oil

The gallic acid equivalent in MSNO was 27.89 ± 1.43%, while it was 53.70 ± 1.17% for OEO. By applying mass balance by component, it was determined that 51.94 wt.% of the MSNO is OEO. This concentration, which indicates that all of the OEO remained in mixture with the A-MSN, corresponds not only to the encapsulated OEO, but also to those adsorbed on the surface of the A-MSN. Therefore, the amount of OEO encapsulated in the A-MSN may be much lower than that added to the mixture, which is in agreement with Rehman et al. [28], who reported 22–48% loading capacity in SBA-15 (Santa Barbara Amorphous-15) mesoporous silica. In addition, 29.12% was reported in APTES functionalized nanotubular mesoporous silica [29], whereas ≈ 30.0–63.7% was reported in A-MSN [30]. According to these results, further experiments were conducted by adding 50% (*w*/*w*) of OEO and A-MSN relative to MSNO, to obtain comparable treatments.

### 2.5. In Vitro Antimicrobial Activity of Active Edible Films

The inhibition zones of AEFs added with MSNO were not uniform, possibly due to the entry of water molecules from the agar, causing expansion of the AEFs and contributing to the release of OEO into the agar because of the hydrophilic character of API [31]. In addition, the diffusion of OEO through the volatilization of its components, could be non-homogeneous. The addition of 9% (*w*/*w*) of MSNO significantly increased (*p* < 0.05) the inhibition of *E. coli* O157:H7 from 11.80 ± 0.79 cm^2^ (control) to 17.66 ± 1.87 cm^2^. This concentration was equivalent to 4.5% OEO (*w*/*w*) according to the aforementioned results, which was higher than previously reported [32]. This was done to ensure good antimicrobial properties against *E. coli*, which ranged 2–3% (*w*/*w*). However, it should be noted that there are discrepancies about the effectiveness of oregano essential oil, due to the variability in the composition [33]. Furthermore, a study on CH-containing films revealed increased antimicrobial activity against various spoilage and foodborne pathogens due to the addition of essential oils [34].

### 2.6. Stability of Film-Forming Solutions

The stability of the film-forming solution of API and CH (44.3 ± 0.2 mV) was slightly increased by the addition of A-MSN (45.9 ± 0.7 mV), probably due to the repulsion between the positively surface-charged A-MSN and the amino acids with positively charged groups. This could be attributed to the pH of the film-forming solutions (3.11), which was below the pH of least solubility reported for API (5.00), which is directly related to the isoelectric point of amaranth proteins [35]. The addition of OEO decreases the stability of the film-forming solution (40.1 ± 0.4 mV) due to its hydrophobic character, but this was counteracted by its encapsulation in A-MSN (MSNO). It should be noted that the film-forming solution of API and CH presented greater stability than the film-forming solution of only API (21.3 ± 0.3 mV), because there is repulsion between CH (protonated) and API (net positive charge) at the final pH of the solutions. The degree of repulsion between adjacent particles in film-forming solutions expressed as ζ potential (mV) is an indicator of their stability, because the particles agglomerate to a lesser extent during solvent evaporation and are homogeneously distributed [36]. Moreover, solutions with ζ potential >|30| mV possess very high stability [37]. This is the case for all film-forming solutions of the different AEFs.

### 2.7. Color, Morphology, and Topography of Active Edible Films

**Figure 3:**
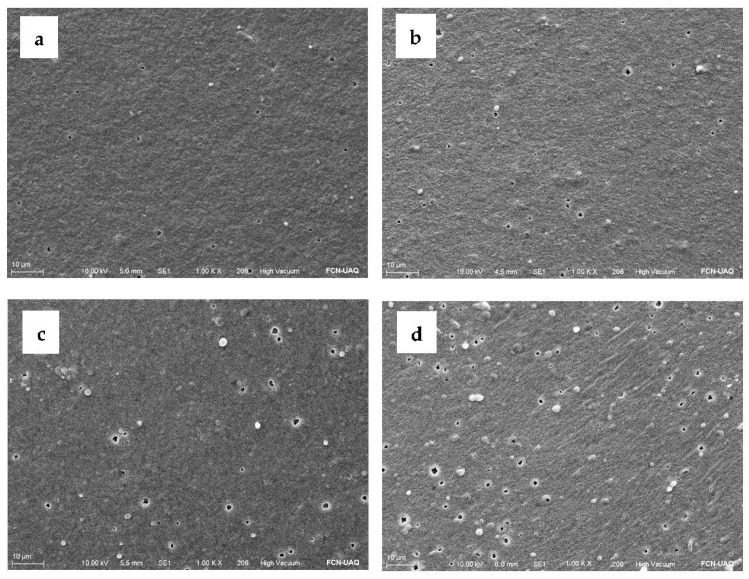
Scanning electron micrograph of active edible films (1000× amplification). (**a**) 4:1 (amaranth protein isolate:chitosan, *w*/*w* ratio); (**b**) 9:1 (amaranth protein isolate:chitosan, *w*/*w* ratio); (**c**) 9:1 (amaranth protein isolate:chitosan, *w*/*w* ratio) + amino-functionalized mesoporous silica nanoparticles (A-MSN); (**d**) 9:1 (amaranth protein isolate:chitosan, *w*/*w* ratio) + mesoporous silica nanoparticles loaded with oregano essential oil (MSNO).

There was no significant difference in color parameters for any treatment. All the formulated AEF showed a yellowish color (L: 37.12 to 38.70; a: −0.32 to −0.5; b: 7.02 to 8.27) and high opacity (59.46–61.79%). According to the ΔE parameter (0.34–1.71), color differences among AEFs with the same formulation were not significant, indicating their homogeneity. Figure 3 shows a micrograph of different AEFs added with A-MSN and MSNO. Figure 3a,b shows a smooth and homogeneous surface and the presence of some agglomerations that increase with increasing protein content in the mixture. This may be due to the agglomeration of a non-soluble fraction of the API, related in turn to an intermediate state of protein denaturation at acid pH. In addition, globulin-*p* (one of the main amaranth proteins) tends to form high molecular weight aggregates and is susceptible to denaturation at pH below 5 [18]. Agglomerates did not increase appreciably with the addition of A-MSN (Figure 3c), but increased with the addition of MSNO (Figure 3d), which was due to AEO loading possessing lower solubility in the film-forming solution of AEFs.

**Figure 4:**
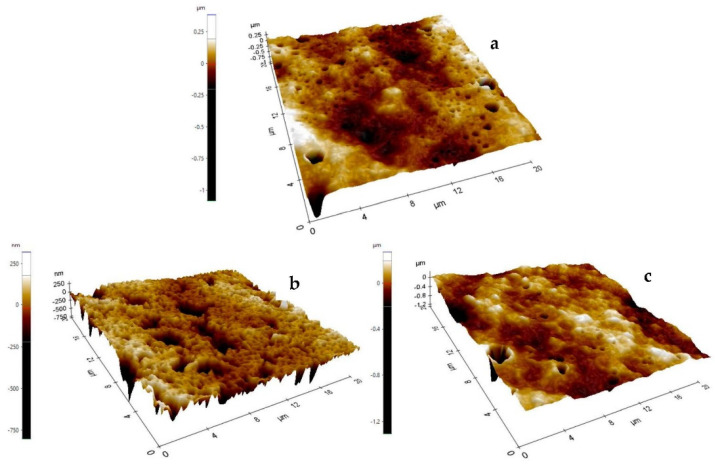
Atomic force micrograph of active edible films 4:1 (amaranth protein isolate:chitosan, *w*/*w*) (**a**) without additions; (**b**) added with emulsified oregano essential oil; (**c**) added with mesoporous silica nanoparticles loaded with oregano essential oil.

Atomic force microscopy (Figure 4) showed that the control AEF presented smooth surface (Ra = 29.400 ± 4.435 nm; Rq = 40.182 ± 7.374 nm), but smoothness significantly decreased (*p* < 0.05) with the addition of emulsified OEO (Ra = 61.073 ± 10.806 nm; Rq = 81.981 ± 14.888 nm). However, addition of MSNO showed similar (*p* > 0.05) roughness as the control (Ra = 33.51 ± 5.639 nm; Rq = 39.476 ± 8.105 nm), and was probably associated to better solubilization of the encapsulated OEO in the polymer matrix.

### 2.8. Thickness, Mechanical Properties, and Water Vapor Permeability (WVP) of Active Edible Films

**Figure 5:**
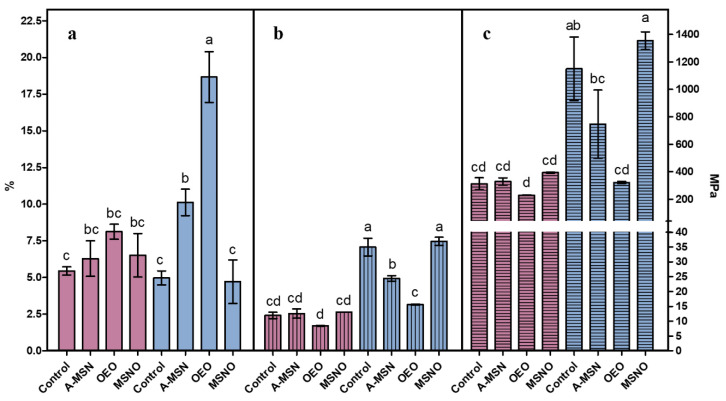
Mechanical properties of active edible films with 9:1 (amaranth protein isolate:chitosan, *w*/*w* ratio) (red) and 4:1 (amaranth protein isolate:chitosan, *w*/*w* ratio) (blue), added with amino-functionalized mesoporous silica nanoparticles (A-MSN), emulsified oregano essential oil (OEO), or mesoporous silica nanoparticles loaded with oregano essential oil (MSNO). (**a**) Elongation (%); (**b**) tensile strength (MPa); (**c**) Young’s modulus (MPa). Columns not connected with the same letter are significantly different (*p* < 0.05).

The addition of different amounts of A-MSN and/or OEO to AEF resulted in different total solids, which did not significantly affect thickness (120.0–166.8 μm). The mechanical properties of the AEFs are shown in Figure 5, which are contrasted below with reported values for elongation, tensile strength, and Young’s modulus of different film materials [38]. Only treatments with 4:1 weight ratio (API:CH) added with A-MSN or emulsified OEO showed different elongation values, with the latter exhibiting 18.66 ± 1.73%. This value is similar to high density polyethylene (20–50%), whereas other treatments tested revealed low elongation, similar to polystyrene (2–3%).

The tensile strength of the 9:1 weight ratio treatments did not show significant differences, presenting values similar to low density polyethylene (7–25 MPa). In the case of the 4:1 treatments added with A-MSN and OEO, the tensile strength was lower than the control, or the MSNO films, whose values are similar to high density polyethylene (19–31 MPa) or polystyrene (31–49 MPa).

From the Young’s modulus results, the AEFs are similar to low density polyethylene (150–340 MPa), except for the API:CH 4:1 control and that added with MSNO that showed values similar to high density polyethylene. Young’s modulus is a property of materials directly related to their stiffness; it denotes the ratio between the longitudinal stress applied to achieve linear deformation of an elastic material, so higher values indicate that more force must be applied to deform the material. Following the experimental design, the factors API:CH, C (type of coating), and their interaction, resulted in significant (*p* < 0.001) results, indicating high interactions among the AEF components.

The higher values of tensile strength and Young’s modulus of AEFs with 4:1 ratio (API:CH; *w*/*w*) than those with the 9:1 ratio are associated to the higher content of CH in the mixture, because the CH control film presented very high values of these properties (732.7 ± 248.3 MPa and 27,169.9 ± 2957.9 MPa, respectively). In addition, the film forming solution of CH and API did not include glycerol, so a higher proportion of CH in the mixture indicates a lower concentration of plasticizer, making the AEF more rigid. This effect was counteracted by the addition of OEO, because these AEF exhibited greater flexibility (higher values of elongation and lower Young’s modulus) showing, in turn, a plasticizing effect of OEO. The hydrophobic characteristic of the essential oil may prevent its chemical interaction with API and CH, being trapped within the polymeric chains interfering with them, and consequently reducing the tensile strength of the films [39]. According to the above data, it was concluded that the 4:1 blend (API:CH, *w*/*w*) produced the best AEF.

The AEFs did not show significant difference in WVP (3.01 × 10^−5^–4.01 × 10^−5^ g/day ∗m∗Pa), which is similar to a previous report on triticale protein containing OEO [40]. However, it has been reported that the addition of a hydrophobic component to the matrix does not ensure WVP reduction [41]. It has been reported that the addition of OEO can even increase WVP due to discontinuities caused in the polymeric network by the OEO, causing loss of film cohesion, and an increase in transport phenomena through it [42]. The WVP values of this work were similar to those of amaranth flour films with a permeability of 1.2 × 10^−5^ g/day∗m∗Pa [43].

### 2.9. Fourier Transform Infrared Spectroscopy (FT-IR)

**Figure A1:**
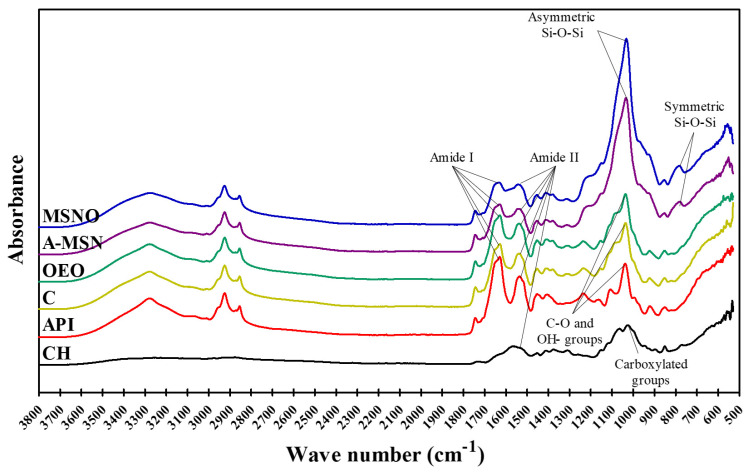
FT-IR spectra of active edible films of chitosan; amaranth protein isolate; amaranth protein isolate:chitosan (4:1 ratio, *w*/*w*): without any addition (C), added with emulsified oregano essential oil (OEO), added with amino-functionalized mesoporous silica nanoparticles (A-MSN), and added with mesoporous silica nanoparticles loaded with oregano essential oil (MSNO).

The chemical interactions between the components of AEFs can be visualized in Figure A1. The AEF spectrum showed peaks corresponding to amide I and amide II, generated by the vibration of peptide groups and related to the secondary structure of polypeptides and proteins.

The amide I band (1600–1700 cm^−1^) is generated by C=O stretching vibration of the peptide group, while the amide II band (1500–1600 cm^−1^) is generated by N-H bending and C-N stretching [44]. A decrease in the absorption peak of both amides of API were identified in the presence of CH, and it was more evident for A-MSN and MSNO. The spectrum of CH, in addition to presenting the characteristic peak of amide II, also showed one peak at 1042–1045 cm^−1^ corresponding to the carboxylated groups associated with its antimicrobial activity [45]. The absorption peak at 1042–1045 cm^−1^ associated with C-O and -OH groups showed decreased intensity for the AEF. This result was probably due to hydrogen bridges between glycerol and hydroxyl groups of the chitosan and API [39,46].

On the other hand, in the spectra of A-MSN- and MSNO AEFs, a strong absorbance at 1037–1039 cm^−1^ and a peak at 800 cm^−1^ are observed. This effect can be associated with a shift of the characteristic peaks of the Si-O-Si asymmetric and symmetric stretching of the SiO_2_ network, respectively [47]. This peak was previously identified by other authors in the range of 1075–1102 cm^−1^ [47,48,49] and could have been shifted due to the interaction of MSN with the polymer matrix and an overlap with the peak at 1042–1045 cm^−1^.

### 2.10. Effect of Edible Coatings on Fresh Beef Shelf Life

#### 2.10.1. Physical and Chemical Properties

**Figure 6:**
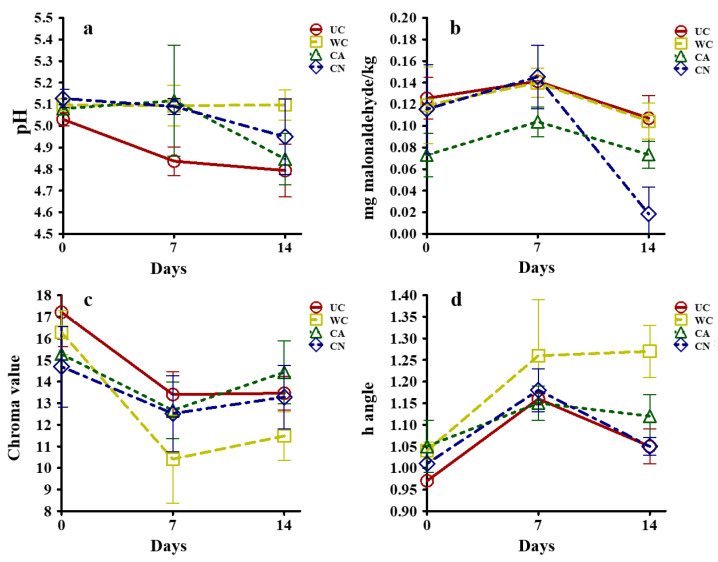
Effect of edible coatings on fresh beef physical and chemical properties. (**a**) Effect on pH; (**b**) Effect on TBARS (mg malonaldehyde/kg meat); (**c**) Effect on chroma value; (**d**) Effect on h-angle. UC: control sample without coating; WC: coated sample without any addition; CA: coated sample added with emulsified oregano essential oil (OEO); CN: coated sample added with mesoporous silica nanoparticles loaded with oregano essential oil (MSNO).

The physical and chemical properties of fresh coated beef are reported in Figure 6. Treatments showed no significant effect on pH values (Figure 6a) (Univar H-F > 0.05). An opposite effect was observed (Univar H-F < 0.05) on TBARS concentration (Figure 6b), as the CN treatment showed antioxidant activity after 14 d of storage, compared to treatments without OEO.

The antioxidant properties of OEO are attributed to its content of phenolic compounds, such as thymol, which constitutes 66.3% of its composition [12]. The hydroxyl groups of the phenolic ring of these compounds can donate a hydrogen atom, nullifying free radicals and consequently, blocking oxidizing compounds formation [50]. For color (Figure 6c,d), only a time effect was observed (Univar H-F < 0.05); however, treatments with emulsified or encapsulated essential oil did not decrease their color saturation (C-value) over time, unlike treatments without added essential oil. This may be attributed to the antioxidant effect of the essential oil that can decrease the formation of metmyoglobin (grayish brown), which accumulates on the surface of the meat [51]. This phenomenon is also promoted by low oxygen concentrations [52] like those produced by vacuum packaging.

#### 2.10.2. Antimicrobial Properties

**Figure 7:**
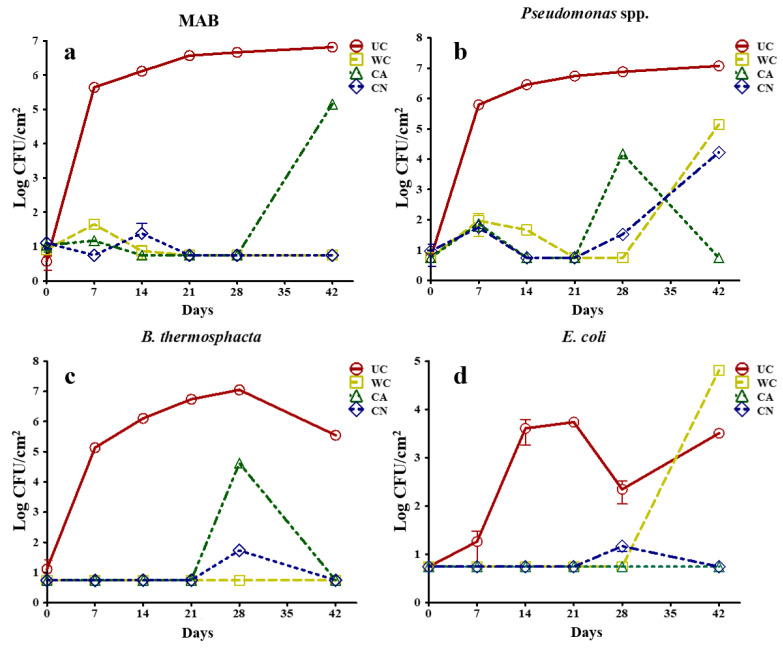
Antimicrobial effect of active edible films on the growth (Log CFU/cm^2^) on different microorganisms in fresh beef. (**a**) MAB; (**b**) *B. thermosphacta*; (**c**) *Pseudomonas*; (**d**) *E. coli*. UC: control sample without coating; WC: coated sample without any addition; CA: coated sample added with emulsified oregano essential oil (OEO); CN: coated sample added with mesoporous silica nanoparticles loaded with oregano essential oil (MSNO).

The AEF exhibited important effects (Univar HF < 0.001) on antimicrobial properties against tested microorganisms. After 21 d of storage, the uncoated sample reached a MAB population of 6.57 ± 0.01 Log CFU/cm^2^ (Figure 7a) and remained close to this value throughout the experiment. Unpleasant odors have been reported to occur when the MAB population reaches about 10^7^ CFU/cm^2^ [53,54]. In contrast, the WC and CN treatments showed insignificant MAB population sizes, while the CA treatment exhibited a population of 5.16 ± 0.05 Log CFU/cm^2^ after 42 d.

As for *Pseudomonas*, the control sample exceeded 6 Log CFU/cm^2^ after 14 d of storage and reached 7.07 ± 0.03 Log CFU/cm^2^ at d 42 (Figure 7b).

*Pseudomonas* metabolizes amino acids as an energy source after glucose depletion. Once the population reaches 10^6–^10^8^ CFU/cm^2^, there is production of sulfur compounds, esters and amines, followed by viscosity and fruity, putrid, sulfurous, and caseous odors [55]. *Pseudomonas* spp. development was observed in the CA treatment at d 28, when the population reached 4.17 ± 0.02 Log CFU/cm^2^, similar to the CN treatment after 42 d (4.22 ± 0.08 Log CFU/cm^2^).

The WC treatment showed the highest population obtained by the AEFs treatments (5.14 ± 0.02 Log CFU/cm^2^) after 42 d of storage. The anticipated microbial growth from the CA treatment may be due to the solubilization and breakdown of the AEF in contact with the moisture of fresh beef, which could be attributed to lower polymeric matrix interactions in the presence of OEO [40]. The plasticizing effect of OEO may lead to a disruption of the coating matrix and consequently to a less compact polymeric structure [33], resulting in faster water diffusion within the AEF [56]. Thus, when the oxygen barrier properties of AEF are impaired, it is less effective in preventing the growth of aerobic bacteria such as *Pseudomonas* spp. [57]. The encapsulation of OEO in MSN prevented this effect for the CN treatment, while preserving the antimicrobial activity exerted by the OEO showing lower *Pseudomonas* population than that observed in the WC treatment after 42 d.

On the other hand, no growth of *B. thermosphacta* was detected in the WC and CN treatments after 42 d of storage. This is in contrast to the UC treatment that exceeded a population of 6 Log CFU/cm^2^ and 7 Log CFU/cm^2^ after 28 and 42 d, respectively. The CA treatment reached 4.62 ± 0.03 Log CFU/cm^2^ after 28 d (Figure 7c). The above mentioned effect may be related to the requirement of *Pseudomonas* spp for oxygen for growth, but *B. thermosphacta* and lactic acid bacteria can also grow and contribute to food spoilage [53]. Although higher effectiveness of essential oils against Gram-positive bacteria (such as *B. thermosphacta*) than against Gram-negative bacteria has been reported [42,58], it also has been found that high protein content in the food matrix may reduce its antimicrobial properties due to binding of some volatile constituents of the essential oil with proteins [58,59].

For *E. coli*, the UC treatment exceeded the population limit established by NOM-194-SSA1-2004 [60] of 3 Log CFU/g after 14 d of storage (Figure 7d). The WC treatment reached the highest population (4.81 ± 0.08 Log CFU/cm^2^) after 42 d, while the OEO treatments did not allow the development of this microorganism, associated to its antimicrobial activity. The antimicrobial activity of OEO against *E. coli* is well known, and it is attributed to the disruption and depolarization of the cytoplasmic membrane promoted by thymol and carvacrol [61].

The amount of OEO used for an average fresh meat cut (20 cm long x 15 cm wide x 5 cm thick) was 722 mg. From the microbiological standpoint, the meat can be deemed as edible after 42 d of refrigerated storage since the addition of edible coatings containing OEO (Figure 7a–c) showed a maximal population of 5 log CFU/cm^2^ with low *E. coli* population (<1.5 Log CFU/cm^2^). Conversely, uncoated meat showed >5 log CFU/cm^2^ for most microorganisms tested after 7 d (Figure 7a–c), while *E. coli* population rose to almost 4 Log CFU/cm^2^ after 14 d (Figure 7d). Therefore, coated meat lasted 35 d more than the uncoated counterparts, which may indicate economic benefits despite the cost of OEO.

### 2.11. Sensory Acceptability

**Figure A2:**
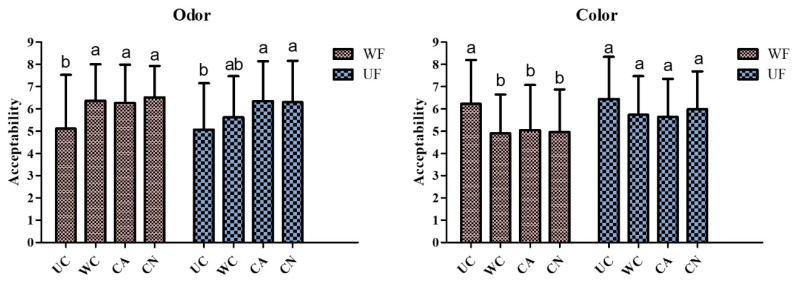
Odor and color acceptability (50 untrained judges) of fresh beef samples stored for 21 d evaluated on treatments using coated samples [API:CH, 4:1 (*w*/*w*)] (WF) or sample coating removal before analysis (UF). UC: control sample without coating; WC: coated sample without any addition; CA: coated sample added with emulsified oregano essential oil (OEO); CN: coated sample added with mesoporous silica nanoparticles loaded with oregano essential oil (MSNO). In the same group (WF or UF) columns not connected by the same letter are significantly different (*p* < 0.05).

The results of the sensory analysis (Figure A2) show that after 21 d of storage, the odor of the coated samples was acceptable, regardless of coating removal before analysis (UF), or not (WF). However, the control sample was rejected, likely due to the presence of unpleasant odors in the control sample due to microbial spoilage.

On the other hand, the visual acceptability of the samples analyzed without removing the coating decreased, irrespective of the additions made to the API:CH (4:1 *w*/*w*) coatings. Removal of the coating (UF) gave a visual acceptability similar to that of the control treatment, which was just acceptable. The type of AEF did not make any difference in relation to acceptability based in odor or in color.

## 3. Materials and Methods

### 3.1. Materials and Culture Media

Chitosan (medium molecular weight 375 kDa, deacetylation 75%), glycerol (≥ 99.5%), D-lactose monohydrate, hydrochloric acid (analytical reagent), cetyltrimethylammonium bromide (CTAB), tetraethylorthosilicate (TeOS), 3-aminopropyltriethoxysilane (APTES), ethanol (>99%, *v*/*v*), 2-thiobarbituric acid (TBA), glycerol, sodium hydroxide, nalidixic acid, hexadecyltrimethylammonium bromide (CTAB), sodium chloride, streptomycin sulfate, thallium acetate and cycloheximide were purchased from Sigma-Aldrich (St. Louis MO, USA). Tween 80, magnesium sulfite heptahydrate, and potassium dibasic phosphate were purchased from J.T. Baker (Estado de Mexico, Mexico). Standard plate count agar, bismuth sulfite agar, nutrient broth, yeast extract, bacteriological agar, and casein peptone were purchased from Bioxon (Cuautitlán, Mexico), while McConkey-sorbitol agar was obtained from Gibco (Mexico City, Mexico). For streptomycin sulfate, thallium acetate, and actidione (STAA) agar, a base with (% *w*/*v*): casein peptone, 2; anhydrous glycerol, 1.5; yeast extract, 0. 2; potassium dibasic phosphate, 0.1; magnesium sulfite heptahydrate, 0.1; bacteriological agar, 1.3 was used; it was supplemented with (% *w*/*v*): streptomycin sulfate, 0.005; thallium acetate, 0.0005; and cycloheximide, 0.0005.

### 3.2. Biological Materials and Microbial Strains

Amaranth (*Amaranthus hypochondriacus*) grains were obtained from the local market in Santiago de Querétaro (Querétaro, Mexico); Mexican oregano (*Lippia graveolens* Kunth) leaves were purchased in Cerrito Parado, Tolimán (Querétaro, Mexico). A specimen was authenticated and deposited in the ethnobotanical collection of the herbarium “Dr. Jerzy Rzedowski” (QMEX), of the Faculty of Natural Sciences at the UAQ (voucher specimen: E. Hernández-Hernández No. 1, Tolimán, Querétaro).

The bacterium used was *Escherichia coli* O157:H7, obtained from the culture collection of the Food Biotechnology Laboratory of the Food Research and Postgraduate Department, Faculty of Chemistry, Universidad Autónoma de Querétaro (UAQ), Mexico. This strain was preserved in sterile skim milk and glycerol at -70 °C and was activated in nutrient broth at 37 °C. Subsequently, the culture was incubated at optimal temperature up to the early stationary phase [1,62].

### 3.3. Oregano (Lippia graveolens Kunth) Essential Oil (OEO) Extraction

The essential oil was extracted from dried Mexican oregano leaves by water–steam distillation in an equipment designed by the Biotechnology Group (UAQ, Mexico). The extracted OEO was dried with anhydrous sodium sulfate, followed by filter-sterilization using a Swinnex unit fitted with a polyvinylidene fluoride membrane (Millipore, Burlington, MA, USA) with pore size of 0.45 µm to finally store it in sealed amber vials at 4 °C; the composition of OEO was previously analyzed and reported [12].

### 3.4. Amaranth Protein Isolate (API) Production

Protein isolation was conducted following the method reported by Condés et al. [63], with some modifications. Amaranth flour (AF) was suspended in distilled water (100 g/L) and adjusted to pH 9.0 with NaOH (1 mol/L). The suspension was stirred for 1 h at room temperature, filtered, and then centrifuged at 10,000× *g* for 20 min at 15 °C. The supernatant was recovered, filtered, adjusted to pH 5.0 with HCl (2 mol/L), and stirred for 1 h at room temperature. The precipitated supernatant was centrifuged at 10,000× *g* for 20 min at 4 °C, and the protein pellet was recovered using a minimal amount of water. The previous solubilization and precipitation procedure was conducted twice. Finally, the API was dried at 40 °C for 48 h, ground and stored in refrigeration.

The protein content (nitrogen × 5.85) of the amaranth flour and API was determined following the Kjeldahl method [64]. The yield of API was determined according to Equation (1):

(1) $$\mathrm{Yield}\;\left(\%\right)=\frac{\mathrm{API}\;\left(g\right)\;}{\mathrm{AF}\;\left(g\right)}\times 100$$

### 3.5. Mesoporous Silica Nanoparticles (MSN), Amino-Functionalized Mesoporous Silica Nanoparticles (A-MSN) and Mesoporous Silica Nanoparticles Loaded with Oregano Essential Oil (MSNO) Synthesis

To produce MSN, CTAB (0.5 g) was dispersed in 240 mL of distilled water with 1.75 mL of NaOH (2 mol/L) by using an ultrasound bath at room temperature. The solution was heated in a water bath to 80 °C, then 2.5 mL of TeOS was added dropwise over 5 min under vigorous agitation and stirring, and conditions were kept constant for 2 h. The solid product was filtered, washed twice with distilled water and ethanol (99%), dried in a fume hood (Labconco, Kansas City MO, USA), and calcinated at 500 °C in a muffle furnace (Thermolyne Scientific, Wetherill Park, NSW, Australia) for 5 h to remove traces of the surfactant [35,65]. The resulting MSN were surface modified by suspending 500 mg in 10 mL of absolute ethanol, then a solution of APTES (100 mg/mL) was added at a ratio of 1 mL/100 mg of MSN, and the final mixture was stirred for 12 h at room temperature. The amino-functionalized MSN (A-MSN) was filtered and washed with absolute ethanol [65]. The size and morphology of the nanoparticles were characterized using a scanning electron microscope (FEI Quanta-250 FEG, Hilsboro, OR, USA) by coating with gold and evaluated using an accelerating voltage of 10 kV. The average size of the A-MSN was determined using image analysis (Image J, version 1.53, https://imagej.nih.gov/ij/download.html accessed on 18 February 2021).

A-MSN were loaded with OEO by dissolving 0.1 g of A-MSN and 0.1 g of OEO in 5 mL of absolute ethanol; the solution was stirred for 24 h in a fume hood (Labconco,) until complete solvent volatilization [66].

### 3.6. Mesoporous Silica Nanoparticles Loading Capacity

To determine the concentration of OEO encapsulated in MSNO, the total phenols concentration in MSNO and OEO was determined, using the Folin–Ciocalteu method [67]. First, 100 mg of either OEO or MSNO were added with 10 mL of absolute ethanol at 25 ± 1 °C protected from light, and under constant agitation for 12 h, followed by centrifugation at 4000× *g* for 10 min and the supernatant was decimally diluted twice with absolute ethanol. A mass balance (eq. of gallic acid) was used to calculate the OEO concentration in the MSNO.

### 3.7. Minimum Inhibitory Concentration (MIC) and Combined Minimum Inhibitory Concentration of Oregano Essential Oil and Chitosan against E. coli O157:H7

The MIC was conducted following the procedure of Hernández-Hernández et al. [1], with some modifications. OEO was added in nutrient broth with Tween 80 (10% *v*/*v*) to produce concentrations of 1–20 mg/mL. Separately, a nutrient broth solution was prepared with CH (1% *w*/*v*), lactic acid (0.050% *v*/*v*) and Tween 80 (10% *v*/*v*); this solution was used to produce 0.25–1.0 mg/mL CH concentrations, keeping same lactic acid concentration. A nutrient broth solution containing 0.5 μL/mL lactic acid, and another containing 10% (*v*/*v*) Tween 80, were used as controls. All concentrations were tested against *E. coli* O157:H7 (10^5^ CFU/mL) in early exponential stage. After 6 h, 18 h, and 24 h incubation times at 37 °C, the population was counted following the drop method [68]. The MIC was defined as the lowest concentration (mg/mL) that completely inhibited *E. coli* O157:H7. This methodology was also conducted to evaluate the combined antimicrobial effect of OEO and CH.

The fractional inhibitory concentration index (FIC_Index_) was calculated as FIC_CH_ + FIC_OEO_, where FIC_CH_ = (MIC_CH_ in combination/MIC_CH_ alone) and FIC_OEO_ = (MIC_OEO_ combined/MIC_OEO_ alone). The antimicrobial interaction may be interpreted as synergistic, additive or antagonic if the FIC_Index_ < 1, = 1, or > 1, respectively [69]. However, Odds [70] proposed a more conservative interpretation of synergistic (FIC_Index_ < 0.5), additive (0.5 ≤ FIC_Index_ ≤ 4) or antagonic (FIC_Index_ > 4) interactions to avoid reproducibility problems.

### 3.8. Preparation and Characterization of Film-Forming Solutions Added with Amino-Functionalized Mesoporous Silica Nanoparticles, Emulsified Oregano Essential Oil or Mesoporous Silica Nanoparticles Loades with Oregano Essential Oil, and Preparation of Active Edible Films (AEF)

The API was prepared as 2% (*w*/*v*) aqueous solution, adjusting to pH = 3.0 with HCl (2 mol/L) and stirred for 1 h at room temperature. The CH solution was obtained by suspending 1% (*w*/*v*) CH in 0.5% (*v*/*v*) lactic acid, stirring for 1 h at 80 °C, and cooled to room temperature. Edible films were prepared from mixtures of API and CH solutions using 2 ratios, 4:1 and 9:1 (API:CH; *w*/*w*), keeping the total soluble matter at 700 mg. The API solution was added to the CH solution under vigorous agitation, glycerol was added as a plasticizer (30% *w*/*w* relative to API) and stirred for 15 min. Then, A-MSN and MSNO solutions (10 mg/mL, pH 3) were placed in an ultrasonic bath for 5 min and added to the film-forming solution. For OEO addition, an emulsion of Tween 80 (5% *v*/*v*), Tween 20 (5% *v*/*v*) and AEO (5% *v*/*v*) was prepared in distilled water at room temperature; the mixture was vortexed before and after the addition of OEO, homogenized in an ultrasonic bath for 5 min, and finally added to the film-forming solution.

The different film-forming solutions were then homogenized (Ultraturrax T25 Basic) for 1 min at 6500 rpm followed by 2 min at 9500 *x g*, and then sonicated at 70% amplitude (VCX 500 Vibra-Cell, Newton, CT, USA) for 1.5 min in cycles of 15 s with resting periods of 5 s. The film-forming solutions were poured into 64 cm^2^ Petri dishes and dried at 38 °C for 15 h, at 50% relative humidity in a climatic chamber (Binder, KBF 15, Tuttlingen, Germany) to obtain the AEF [1,35].

#### 3.8.1. Thickness, Mechanical Properties, and Water Vapor Permeability (WVP) of Active Edible Films

Five thickness measurements were performed at randomly selected points of the AEF with a digital micrometer (Mitutoyo, MDC-Lite, Series 293, IL, USA) [71].

The mechanical properties were evaluated using a two factors experimental design: the API:CH ratio of film-forming solution at two levels, 9:1 and 4:1 (*w*/*w*), and type of coating “C” in 4 levels, considering the addition of A-MSN (3% *w*/*w*), OEO (3% *w*/*w*), MSNO (6% *w*/*w*), and a control treatment without any additions. The response variables were tensile strength, elongation at break, and Young’s modulus, measured using the Instron Universal equipment (Mod. 5543A, Norwood, MA, USA) according to ASTM D882-10 [72]. The films were cut into strips of 1 cm × 5 cm and previously conditioned at 50% relative humidity (RH) and 23 ± 1 °C in an environmental chamber for 24 h. The initial grip gap was programmed at 90 mm and the strips were stretched at a speed of 30 mm min^−1^ until breakage [71,73].

The WVP of the AEF was determined using a gravimetric method, modified from ASTM:E96-00 [74] based on Fick’s law. The AEFs were cut and placed in 5 cm glass jars with an internal diameter opening of 3.5 cm and distilled water inside (RH_1_= 100%) and a perforated screw-on lid that allowed an exposure area of 9.079 × 10^−4^ m^2^. The AEFs were placed between two Teflon gaskets to ensure a hermetic seal. Once assembled, the cells were placed inside a permeability chamber with silica gel (HR_2_ ≈ 0%) on the plate of an analytical balance. The water vapor that diffused through the AEFs was absorbed by the atmosphere created. The weight of the cells was automatically monitored at 1 min time intervals for 11 h at a controlled temperature of 30 °C. WVP was calculated using Equations (2) and (3):

(2) WVP=GtAxΔP

From where:

(3) $$\Delta P=\frac{P^{*}}{100}\left({\mathrm{HR}}_{2}-{\mathrm{HR}}_{1}\right)$$

where: (G/t)/A = Permeance; G/t: slope of the graph of weight loss versus time; x = thickness; A = film area; P* = vapor pressure of pure water at 30 °C; HR_1_ = relative humidity inside the cell; HR_2_ = relative humidity outside the cell; ΔP = vapor pressure difference.

#### 3.8.2. Color, Morphology and Topography of Active Edible Films

A colorimeter (Konica, Minolta, Osaka, Japan) was used to evaluate the parameters L* (lightness), a* (green-red), and b* (yellow-blue) at three randomly selected points for each film. In the same way, the L* parameter of the films on black (BB) and white background (WB), was determined for the calculation of opacity according to Equation (4):

(4) $$\mathrm{Opacity}=\frac{{L\;*}_{\mathrm{BB}}}{{L\;*}_{\mathrm{WB}}}\times 100$$

The morphology of the edible films was studied by scanning electron microscopy following the method indicated in Section 3.5. Micrographs with magnification up to 10,000× were obtained from the following: AEF API:CH (*w*/*w*): 4:1; 9:1; 9:1+A-MSN (3% *w*/*w*); 9:1+MSNO (6% *w*/*w*); and MSNO solution (10 mg/mL).

Topography of AEF was observed following the method reported by Escamilla-García et al. [75]. An atomic force microscope (Multimode V, Veeco, CA, USA) was used, applying the tapping method using silicon probes (RTESP Bruker cantilevers, Santa Barbara, CA, USA). Samples of all AEF of 0.5 × 0.5 cm were scanned at 256 × 256 pixels resolution.

#### 3.8.3. Fourier Transform Infrared Spectroscopy (FT-IR)

The chemical interaction among CH, API, and MSNO, was analyzed by Fourier transform infrared spectroscopy (FT-IR) (LabRAM IR2, Horiba Jobin Yvon, Kyoto, Japan). Spectra of AEFs were obtained in the region between 4000–600 cm^−1^, with a resolution of 4 cm^−1^.

#### 3.8.4. In Vitro Antimicrobial Activity of Active Edible Filsm

The in vitro antimicrobial activity was determined employing the agar disc diffusion method [60], with some modifications. The API:CH 4:1 (*w*/*w*) was produced by adding increasing amounts of MSNO (0–12% *w*/*w*) and cut into 2.5 mm diameter circles. An amount of 10 mL of soft nutrient agar (0.85% *w*/*v*) previously inoculated with 10^5^ CFU/mL of *E. coli* O157:H7 and poured over 15 mL of nutrient agar (1.5% *w*/*v*). Then, discs of AEF were placed on the surface, followed by incubation at 37 °C for 24 h. The inhibition area against *E. coli* O157:H7 was measured using the ImageJ v. 1.53 analysis software.

#### 3.8.5. Active Edible Films Effect on Fresh Beef

An experimental design of repeated measures over time was applied using one factor with four levels: (1) control without coating (UC), (2) AEF coated without addition of OEO or MSNO (WC), (3) AEF coated added with emulsified OEO (6% *w*/*w*) (CA), and (4) AEF coated added with MSNO (12% *w*/*w*) (CN). The response variables, pH, TBARS, and color, were evaluated at 0, 7, and 14 d, while the antimicrobial activity against *E. coli*, *Pseudomonas* spp., *Brochothix termosphacta* and mesophilic aerobic bacteria (MAB) were evaluated at 0, 7, 14, 21, 28, 35, and 42 d. Samples were prepared from a whole piece of fresh sirloin purchased at a local market in Santiago de Querétaro (Mexico) and transported immediately under refrigeration to the laboratory. The meat surface was sprayed with ethanol (70% *v*/*v*) and flamed with a blowtorch; the burnt part of the meat was removed with a sterile knife without removing more than 0.5 cm of surface thickness, and 1 cm thick square pieces of 3 × 3 cm^2^ were cut. For the antimicrobial analysis, treatments were randomly applied as follows: the different films were previously prepared on Petri dishes of 55 mm in diameter; two films were aseptically placed on both sides of the meat sample. The samples were vacuum packed (50.7 kPa) in Food Saver V3800 bags (Antwerp, New York, USA) of low-density multilayer polyethylene, and finally stored at 4 °C. To evaluate the antimicrobial properties of films, the microbiota of both sides of the meat surface was recovered using a sterile swab moistened in sterile saline solution (0.85% NaCl, *w*/*v*), followed by submerging in 10 mL of this solution. Decimal dilutions were added to plates using the appropriate media for each strain: plate count agar for MAB, STAA agar for *B. thermosphacta*, nutrient agar added with 2% (*w*/*v*) sucrose for *Pseudomonas*, and McConkey-sorbitol agar for *E. coli* O157:H7. Microbial populations were quantified by the surface spreading method (100 µL dispersion on the agar surface), except for mesophilic aerobic bacteria, for which the pour plate technique was used. Plates were incubated for 48 h at 30 °C for *B. thermosphacta* and *Pseudomonas,* and at 37 °C for *E. coli* O157:H7 and MAB.

For the physicochemical analyses, film-forming solutions were prepared and randomly distributed on meat samples at a rate of 0.06 mL/cm^2^ using a sterile glass rod, after which they were left to dry in a laminar flow cabinet for 30 min prior to a second application. The procedure was repeated for the other side of the sample. Determination of lipid oxidation was performed by homogenizing 0.50 ± 0.01 g of meat in 2.5 mL of TBA solution (0.375% *w/v* TBA, 15% *w/v* trichloroacetic acid and 0.25 N HCl). The homogenate was transferred to a boiling water bath for 10 min and subsequently cooled in an ice bath. The sample was centrifuged at 4000× *g* for 10 min at 4 °C, and the absorbance of the supernatant was measured at 532 nm (A_532_) in a spectrophotometer (Thermo Scientific). The TBARS (thiobarbituric acid reactive substances) concentration was ere calculated according to Equation (5) [1]:

(5) $$\mathrm{TBARS}\;\left(\frac{\mathrm{malonaldehyde}\;\mathrm{mg}}{\mathrm{kg}\;\mathrm{of}\;\mathrm{meat}}\right)=A_{532}\times 2.77$$

The pH of the beef samples was determined using an Orion Star A211 potentiometer (Thermo Scientific). A sample of 10 g was homogenized in 90 mL of distilled water [76]. The color of meat samples was obtained according to Section 3.8.2, and the chroma coordinate (C*) and hue angle (h*) were determined according to Equations (6) and (7).

(6) $$C^{*}={\left({a\;*}^{2}+{b\;*}^{2}\right)}^{1/2}$$

(7) $$h^{*}=\tan^{-1}\;\left(b\;*/a\;*\right)$$

#### 3.8.6. Sensory Evaluation

Sensory evaluation was carried out to evaluate the odor and color of fresh meat coated with the different AEF after 21 d of refrigerated storage (4 °C ± 2 °C) by 50 untrained judges. A 9-point descriptive hedonic scale was used for evaluation, where 1 = extremely undesirable; 2 = very undesirable, 3 = moderately undesirable; 4 = slightly undesirable, 5 = neither acceptable nor rejectable; 6 = slightly desirable; 7 = moderately desirable; 8 = very desirable and 9 = extremely desirable. An average score <6 was determined to indicate an unacceptable meat sample [77]. A second sensory evaluation was applied to determine the effect of AEF removal on the acceptability of previously coated fresh meat following a similar procedure.

### 3.9. Statistical Analysis

All determinations were conducted in triplicate and the mean ± standard deviation (SD) was reported. All comparisons were conducted using the Tukey test (*p <* 0.05).

Statistical analyses corresponding to the repeated measures design used here, consist of using Mauchly’s test to assess the so-called sphericity assumption. If this assumption were not adequate, a corrected analysis of variance was carried out according to the Huynh–Feldt criterion to calculate *p*-values.

## 4. Conclusions

The encapsulation of OEO in MSN successfully achieved the preservation of its antioxidant and antimicrobial properties and reduced its destabilizing effect on the edible active film matrix. The designed AEF produced a clear increase in the shelf life of fresh beef stored under refrigeration, inhibiting the development of pathogenic and spoilage microorganisms for 42 d, while achieving consumer acceptability for up to 21 d; thus, demonstrating its potential use in the preservation of this type of products. Research is needed to determine the synergistic mechanism of the essential oil in combination with CH, as well as the determination of the specific inhibition mechanisms of spoilage and pathogenic foodborne microorganisms. In addition, further research is required to ensure the safety of consuming nanoparticles-containing coating materials.

## Figures and Tables

**Table 1:** Protein concentration (%P; N × 5.85) on dry basis and extraction yield of amaranth protein isolates.

AF Protein (%)	API Protein (%)	Yield (%)	Reference
13.98 ± 0.17	61.34 ± 0.45	19.71 ± 2.46	-
14.6 ± 2.1	85.42 ± 1.26	12.78	Das et al. [15]
-	85.13 ± 0.01	-	Cortez-Trejo et al. [16]

Mean (*n* = 3) ± S.D. AF: amaranth flour; API: amaranth protein isolate.
